# Secondary C1q Deficiency in Activated PI3Kδ Syndrome Type 2

**DOI:** 10.3389/fimmu.2019.02589

**Published:** 2019-11-11

**Authors:** Ying Hong, Sira Nanthapisal, Ebun Omoyinmi, Peter Olbrich, Olaf Neth, Carsten Speckmann, Jose Manuel Lucena, Kimberly Gilmour, Austen Worth, Genomics England Research Consortium, J. C. Ambrose, Nigel Klein, Despina Eleftheriou, Paul Brogan

**Affiliations:** (1) Genomics England, London, UK; (2) William Harvey Research Institute, Queen Mary University of London, London, EC1M 6BQ, UK; ^1^Infection, Immunology and Inflammation Research & Teaching Department, University College London Great Ormond Street Institute of Child Health, London, United Kingdom; ^2^Department of Pediatrics, Thammasat University, Bangkok, Thailand; ^3^Paediatric Infectious Diseases, Rheumatology and Immunology Unit, Institut of Biomedicine of Seville, Hospital Universitario Virgen del Rocío, Seville, Spain; ^4^Faculty of Medicine, Center for Chronic Immunodeficiency, Medical Center, University of Freiburg, Freiburg im Breisgau, Germany; ^5^Unidad de Inmunología, Hospital Universitario Virgen del Rocio, Seville, Spain; ^6^Clinical Immunology Laboratory, Great Ormond Street Hospital NHS Foundation Trust, London, United Kingdom; ^7^ARUK Centre for Adolescent Rheumatology, UCL, London, United Kingdom

**Keywords:** digital vasculitis, C1q deficiency, SHORT syndrome, activated PI3Kδ syndrome type 2, hyper-IgM syndrome, immunodeficiency

## Abstract

Monogenic forms of vasculitis are rare but increasingly recognized. Furthermore, genetic immunodeficiency is increasingly associated with inflammatory immune dysregulatory features, including vasculitis. This case report describes a child of non-consanguineous parents who presented with chronic digital vasculitis early in life, is of short stature, has facial dysmorphia, immunodeficiency (low serum IgA, high serum IgM), recurrent bacterial infections, lymphoproliferation, absence of detectable serum C1q, and low classical complement pathway activity. We identified a previously reported *de novo* heterozygous pathogenic splice mutation in *PIK3R1* (c.1425 + 1G > A), resulting in the skipping of exon 11 of the p85α subunit of phosphatidylinositol 3-kinase and causing activated PI3Kδ syndrome type II (APDS2). This explained the phenotype, with the exception of digital vasculitis and C1q deficiency, which have never been described in association with APDS2. No mutations were identified in *C1QA, B*, or *C*, their promoter regions, or in any other complement component. Functional studies indicated normal monocytic C1q production and release, suggesting that the observed C1q deficiency was caused by peripheral consumption of C1q. Since C1q deficiency has never been associated with APDS2, we assessed C1q levels in two unrelated patients with genetically confirmed APDS2 and confirmed C1q deficiency in those two cases as well. This observation suggests C1q deficiency to be an inherent but previously unrecognized feature of APDS2. We speculate that the consumption of C1q is driven by increased apoptotic bodies derived from immune cellular senescence, combined with elevated IgM production (both inherent features of APDS2). Secondary C1q deficiency in APDS2 may further contribute to immunodeficiency and could also be associated with inflammatory immune dysregulatory phenotypes, such as the digital vasculitis observed in our case.

## Background

The introduction of next-generation genetic sequencing (NGS) into routine clinical care has resulted in a significant diagnostic impact in any fields of medicine, including primary immunodeficiency, autoinflammation, and vasculitis (1). Indeed, many different forms of monogenic vasculitis are increasingly described (2–7) and, though rare overall, are important to detect as targeted treatment is sometimes possible; this is an important tenet of precision medicine. It is also increasingly recognized that inflammatory features occur in primary immunodeficiencies due to immune dysregulation. This poses particular therapeutic challenges since treatment of these autoinflammatory features often requires immunosuppression; this is of particular concern in patients with coexisting immunodeficiency. This clinical problem is particularly acute in the absence of a confirmed molecular diagnosis, and this argues strongly for the use of NGS early in the diagnostic pathway for such patients. Not only has NGS clearly impacted on the discovery of new genetic immunological diseases, but it is also revealing patients with atypical immunophenotypes driven by mutations in known immunodeficiency genes. Herein, we describe a child of non-consanguineous parents who presented with digital vasculitis from early on in life, C1q deficiency, immunodeficiency, recurrent infections, short stature, and facial dysmorphia. Whole genome sequencing revealed a previously reported (8–10) *de novo* heterozygous pathogenic splice mutation in a mutation hotspot of *PIK3R1* (c.1425 + 1G > A), resulting in the skipping of exon 11 of the p85α subunit of phosphatidylinositol 3-kinase and confirming the diagnosis of activated PI3Kδ syndrome type II (APDS2). This fully explained the phenotype, though with the exception of C1q deficiency, which has never been described in association with APDS2. Confirmation of low C1q in two unrelated cases of APDS2, and functional studies confirming normal production and release of C1q from monocytes from the index case, suggested that secondary consumptive C1q deficiency is an inherent but previously unrecognized feature of APDS2, which may contribute to immune dysregulation associated with that primary immunodeficiency.

## Case Presentation

**Figure 1:**
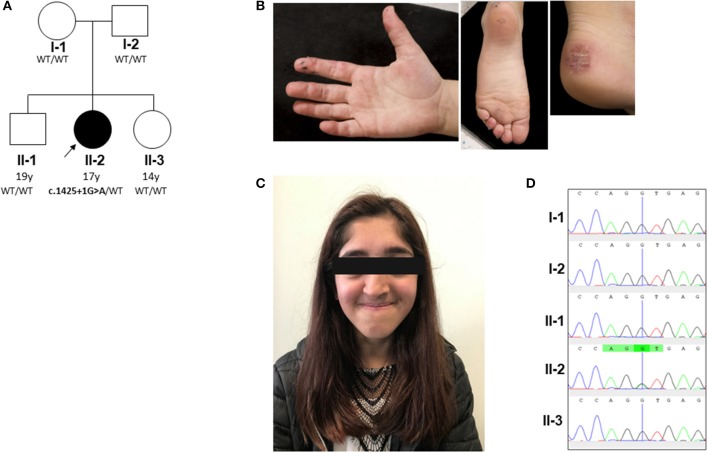
Pedigree and clinical phenotype of proband. **(A)** Pedigree, with affected individual (the proband) in black and unaffected individuals in white. *PIK3R1* genotyping of the pedigree identified a *de novo* mutation in the proband; WT, wild type. **(B)** Peripheral vasculitis of digits and heel. **(C)** Facial gestalt reminiscent of SHORT syndrome (**s**hort stature, **h**yperextensibility of joints and/or inguinal herniae, **o**cular depression, **r**ieger anomaly of the eye, and **t**eething problems): ocular depression, nasal deviation, and prominent mandible present in the proband (see main text and Supplemental Table 1). **(D)** Sanger sequencing confirmed a heterozygous splice site G/A mutation (black/green overlapping line) at position c.1425 + 1 of *PIK3R1* gene in family member II-2 only. This mutation is absent (single black line corresponding to wild type “G” allele) in the other family members (I-1, I-2, II-1, and II-3).

The proband is currently a 17-years-old female of mixed Indian (mother) and Caucasian (father) ethnicity. Both parents were well with no past medical history of note, as were her two siblings. The pedigree is summarized in Figure 1A. She was referred at the age of 6 years for management and further work up of digital vasculitis on the background of previously documented C1q and IgA deficiency. She had spontaneously developed vasculitic lesions of the fingers, toes, and heels (Figure 1B) at the age of 6 years, with no obvious infectious, cold-exposure, or other trigger. Peripheral pulses were intact, blood pressure was normal, there were no urinary symptoms or any history of hematuria or proteinuria, and no other clinical features of systemic vasculitis were present. Her past medical history was remarkable, with intrauterine growth retardation and short stature: age 17 years; height 152 cm (second percentile); weight 38.6 Kg (<<0.4th centile); facial dysmorphia with deep-set eyes, astigmatism prominent jaw, and unilateral choanal atresia (Figure 1C). Throughout childhood, multiple significant infections were documented: frequent (six episodes per year) sinopulmonary infections starting from the first year of life (presumed bacterial, but no organism isolated by the local hospital); recurrent (at least six episodes per year) otitis media with secondary hearing loss requiring hearing aids; recurrent tonsillitis (number not specified) responding to antibiotics (but no bacterial cause reliably documented); bronchiectasis (age 10 years); gingivitis; an episode of necrotizing cervical lymphadenitis (requiring surgical drainage and intravenous antibiotics (aged 15 years). There was, however, no histological evidence of lymphoma. Other medical issues included gastro-esophageal reflux requiring fundoplication (age 6 years) and obstructive sleep apnea requiring home-administered continuous positive airway pressure ventilation (CPAP; clinical features summarized in Supplemental Table 1).

Laboratory investigations are summarized in Supplemental Table 1. In summary, these revealed elevation of acute phase reactants only during episodes of infection, which normalized in between infections; normal total lymphocyte count; low CD19 B cell count (0.18 × 10^9^/L); modestly reversed CD4: CD8 ratio. IgA levels were consistently below the lower limit of detection and IgM was consistently elevated (3.33 g/L; reference range [RR] 0.5–2.0). Vaccine responses to *C. tetani* and *H. influenzae* B were normal. Serum C1q levels were below the limits of the assay: <13 mg/L (RR 50–250), measured on five separate occasions over ~10 years; classical complement pathway activity was reduced (24%; RR >40%, but not absent as might be expected in primary C1q deficiency); alternative complement pathway activity was normal (99%, RR >10%). Anti-C1q antibodies were negative and serum C2, C3c, and C4 levels were normal. Antinuclear antibodies were moderately positive (1:640); other autoantibodies (including against double-stranded deoxyribonucleic acid, and antineutrophil cytoplasmic antibodies) were negative. A screening for Epstein-Barr virus became positive at the age of 17 years (EBV viral load 2,180 copies/ml of whole blood), although there were no clinical features of acute EBV infection; peripheral blood cytomegalovirus (CMV) viral load was negative. Histology of cervical lymphadenopathy did not reveal any evidence of lymphoma, nor any evidence of EBV infection. A full histological description is provided in Supplemental Table 1.

The clinical diagnosis was therefore that of digital vasculitis from C1q deficiency (presumed genetic) with concomitant (and incidental) selective IgA deficiency. Elevated IgM was initially considered (erroneously, as revealed by genetic testing) a non-specific inflammatory feature. Treatment focused on the management of infections with antibiotics and subsequently antibiotic prophylaxis for infection prevention. Treatment of the digital vasculitis in the absence of any other organ threatening vasculitis included corticosteroids (mainly oral prednisolone, but also high dose intravenous pulsed methylprednisolone for acute flares), hydroxychloroquine (5–6 mg/kg/day orally), azathioprine (2 mg/kg/day), and intermittent use of vasodilators (amlodipine 5 mg/day) to improve digital perfusion. These treatments resulted in some (albeit temporary) therapeutic relief from painful digital vasculitis but never fully resolved this chronic problem. Other treatments and interventions are summarized in Supplemental Table 1.

Both parents and her two siblings displayed no clinical features. Baseline laboratory investigations in those individuals (acute phase reactants; full blood count including differential white cell count; routine clinical chemistry; C1q, C3, and C4 levels) were normal (Supplemental Table 1). There was no evidence of IgA deficiency or elevated IgM in either of the unaffected siblings; immunoglobin levels were not examined in the parents.

## Genetic Testing, Functional Studies of C1q, and Subsequent Clinical Progress

All experimental work was performed with ethical approval (ethics number: 08H071382) and with written informed consent from all adult participants, assent (where appropriate) for children, and parental consent for children. The proband formally re-consented when she reached the age of 16 years.

Initial targeted genetic testing of the proband undertaken as part of routine clinical care (prior to the more widespread introduction of next-generation genetic sequencing) focused on the C1q deficiency as that aspect of the phenotype seemed particularly pertinent to work up of the digital vasculitis. Conventional Sanger sequencing of *C1QA, C1QB*, and *C1QC* was performed, with primers designed using Primer-blast. Supplemental Table 2 shows the forward and reverse primers used to probe each exon of these genes as well as the coverage of primers relative to the start and end position on chromosome 1 (genome reference consortium human build 38 patch 7; CRCh38.p7). Sanger sequencing did not identify any mutations in *C1QA, C1QB*, or *C1QC*. To further investigate the possibility of mutations in non-coding regions that may disrupt RNA transcription (11), complimentary deoxyribonucleic acid (cDNA) was also sequenced using Sanger sequencing (primers provided in Supplemental Table 3) with no mutation found (data not shown). A quantitative polymerase chain reaction of messenger RNA (mRNA) indicated that mRNA expression for all C1q genes (primers provided in Supplemental Table 4) in the proband was 2- to 6-fold higher than in three healthy controls (data not shown). In summary, these initial genetic investigations did not reveal any mutation in C1q to account for the profound and persistent C1q deficiency observed, thus prompting more detailed studies of leukocyte C1q production and release.

## Detection of C1q Production in Macrophages and Peripheral Blood Mononuclear Cells

**Figure 2:**
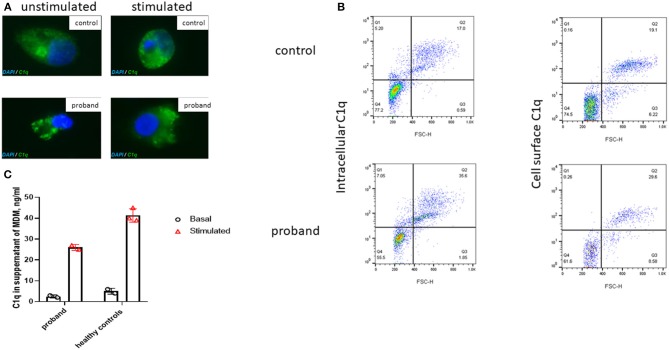
Immunofluorescence microscopy of intracellular C1q in M2 macrophages derived from the proband and a healthy control. **(A)** Immunofluorescent microscopy images taken, using confocal microscopy (original magnification 63×; Supplemental Methods), of unstimulated and stimulated permeabilized macrophages (72 h with 10 μM of dexamethasone and 200 ng/ml of gamma interferon, IFN-γ) from the proband and a healthy control stained with C1q (green) and DAPI (blue). **(B)** Flow cytometry (see Supplemental Methods) demonstrated comparable intracellular and surface C1q staining from a healthy control and the proband monocytes following stimulation with dexamethasone and IFN-γ for 72 h; FSC-H, forward scatter height. **(C)** C1q levels in the supernatant of monocyte derived macrophages (MDM) from the proband and healthy controls before and after stimulation with dexamethasone and IFN-γ for 72 h (as described above). Cultured macrophages from the proband released comparable amounts of C1q into supernatants as healthy controls. Bar chart indicates mean of duplicate measurements in proband and three healthy controls; error bars indicate standard error of mean.

C1q is predominantly produced by cells of monocytic lineage, such as immature dendritic cells and macrophages (12). Monocytes or monocyte-derived macrophages (MDM) were stimulated with 200 ng/ml IFN-γ and 10 μM dexamethasone for 72 h (see Supplemental Methods). Intracellular C1q staining of macrophages was clearly demonstrated in the proband and controls at baseline and following stimulation (Figure 2A). This observation was confirmed using flow cytometry (Supplemental Methods), which also demonstrated comparable intracellular and cell surface C1q staining (Figure 2B) of monocytes in the proband compared with healthy control. Lastly, cultured macrophages from the proband released comparable amounts of C1q into supernatants as healthy controls (Figure 2C). Taken together with the C1q genotyping studies, these results indicated that the cause of the C1q deficiency in the proband was due to peripheral consumption, rather than a defect of production or release, thus indicating that genetic screening needed to be extended beyond the complement system.

## Whole Genome Sequencing Identification of *de novo PIK3R1* Mutation

To further investigate a genetic cause of the atypical clinical and immunological phenotype, the proband and her parents were recruited to the Genomics England 100,000 Genomes Project (protocol version 4, 2017), with written informed consent from parents and assent from the proband; the full protocol is available online at https://doi.org/10.6084/m9.figshare.4530893.v4. Simultaneously, NGS was undertaken using our previously described targeted gene panel for vasculitis and inflammation (1) to mitigate against any potential delay in return of results from whole genome sequencing. Whole genome sequencing revealed a *de novo* class five mutation in *PIK3R1* in the proband: (c.1425 + 1G > A), resulting in the skipping of exon 11 of the p85α subunit of phosphatidylinositol 3-kinase and confirming the diagnosis of activated PI3Kδ syndrome type II (APDS2) (13, 14). This explained the phenotype of absent IgA, elevated IgM, recurrent infections, lymphoproliferation, and dysmorphic features reminiscent of SHORT syndrome (**s**hort stature, **h**yperextensibility of joints and/or inguinal herniae, **o**cular depression, **r**ieger anomaly of the eye, and **t**eething problems), which can also be associated with diabetes and insulin resistance in APDS2 (8, 15). This mutation was also confirmed as the only pathogenic mutation found in the proband using our targeted gene panel (for a full list of genes included, see Supplemental Table 5), which also confirmed that neither parent had the mutation. Sanger sequencing was also then used to further (and definitively) confirm this as a *de novo* mutation in the proband and also confirm its absence in the parents and the two unaffected siblings (Figure 1D). Further in-depth immunophenotyping of the proband confirmed low levels (for her age) of naïve T cells and relatively high percentage of memory CD8 cells, consistent with APDS2 (Supplemental Table 6). C1q deficiency has never been described in association with APDS2 or APDS1, however. This prompted us to contact the authors (PO, JML and ON) of a report describing two previous cases of APDS2 (9), one of whom had the same *PIK3R1* c.1425 + 1G > A mutation as the proband. Serum C1q levels in these two patients were also found to be low. Patient P1 (Supplemental Table 1) had a C1q level of 60 mg/L (RR 170–430; measured using nephelometry) prior to sirolimus treatment. This normalized transiently to 180 mg/L when it was rechecked after >2 years sirolimus, but subsequently fell again when measured on two separate occasions (to 43 and 24 mg/L) despite adequate serum levels of sirolimus. Patient P2 only had the serum C1q level measured whilst on long-term sirolimus treatment: C1q level 21.2 mg/L (RR 170–430). These results indicated that consumptive C1q deficiency may be an integral, but hitherto unrecognized, feature of ADPS2.

Following the confirmation of APDS2 as the diagnosis, the proband was commenced on sirolimus (2 mg/m^2^/day, aiming for pre-dose serum levels between 8 and 12 ng/ml; level of 14 ng/ml obtained) and subcutaneous immunoglobin replacement therapy (hizentra 6 g/week). She remains on antibiotic and anti-fungal prophylaxis (azithromycin, co-trimoxazole, and fluconazole all at standard doses) and took hydroxychloroquine 200 mg once daily. Sirolimus was poorly tolerated and stopped after 3 months due to poor appetite, weight loss, abdominal pain, and oral ulceration. Consequently, we did not have the opportunity to measure C1q levels in response to sirolimus. At the time of writing, she is being worked up for allogeneic hematopoietic stem cell transplantation (allo-HSCT) (8) since the patient has declined further experimental targeted treatment with the PI3Kδ inhibitor, leniolisib (8, 16).

## Discussion

**Figure 3:**
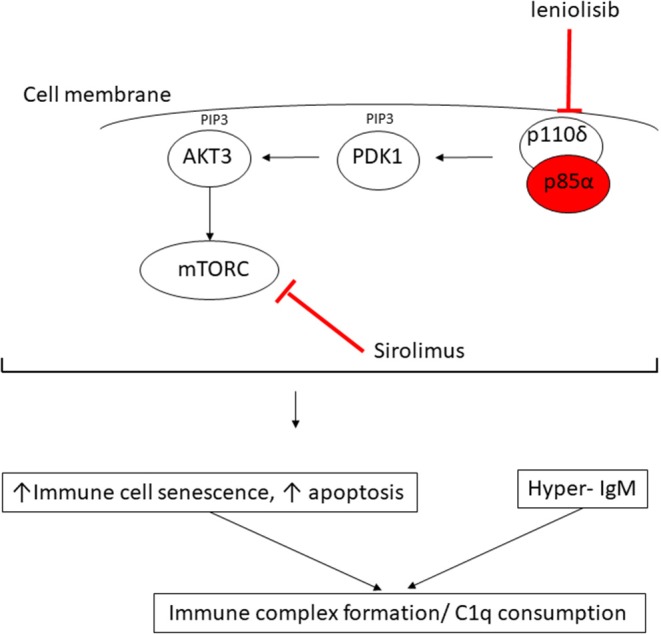
Proposed mechanism of consumptive C1q deficiency in APDS2. Mutated p85α subunit (red oval) of the PI3K complex results in impaired inhibitory contact with the p110δ subunit, with constitutive signaling via the major molecules downstream of the phosphatidylinositol 3-kinase (PI3K) complex: PDK1, phosphoinositide-dependent kinase-1; AKT3, AKT serine/threonine kinase 3; PIP3, phosphatidylinositol (3–5)-trisphosphate; mTORC, mechanistic target of rapamycin complex. Sirolimus exerts its therapeutic effect by blocking mTORC (red line) downstream of the activated PI3K complex; leniolisib directly inhibits the p110δ subunit of the PI3K complex (see main text).

In this report, we provide the first description of C1q deficiency occurring in association with genetically confirmed APDS2 caused by a previously well-described *de novo* splice mutation in *PIK3R1*, c.1425 + 1G > A, which is a mutation hot spot that accounts for 79% of the 64 patients with APDS2 reported to date (8). *PI3KR1* encodes three protein isoforms: the p85α, p55α, and p50α regulatory phosphoinositide 3-kinase (PI3K) subunits. The c.1425 + 1G > A splice site mutation results in skipping of exon 11(delE11) (8, 9, 17), which results in reduction of the normal regulatory inhibitory binding of mutated p85α subunit protein to the p110δ subunit. This results in the constitutive hyperactivation of PI3K (Figure 3) (17) and the clinical phenotype of APDS2 in humans. As our case illustrates, patients with APDS2 typically present with recurrent sinopulmonary infections, bronchiectasis and lymphoproliferation, exhibit hyperactive PI3K signaling, and have a skewing of CD8+ T cells toward terminally differentiated senescent effector cells with short telomeres (8, 17). The B cell immunophenotype includes impaired immunoglobulin class-switch recombination and impaired B cell memory generation and function, hypogammaglobulinaemia, elevated IgM, and a progressive loss of B cells (8). Impaired virus control of EBV and CMV, lymphoproliferation, and various autoimmune phenomena including cytopenias, and enteropathy are also well-described in APDS2 (8). Susceptibility to lymphoid malignancies are a particular concern for patients with APDS2 and APDS1: in a recent international systematic review, the overall rate of malignancy in 243 APDS patients (179 with APDS1 and 64 with APDS2) was 12.8%, with diffuse large B cell lymphoma being the most common, accounting for 89% of malignancies in that series (8). Some patients with APDS2 also have features of SHORT syndrome (**s**hort stature, **h**yperextensibility of joints and/or inguinal herniae, **o**cular depression, **r**ieger anomaly of the eye, and **t**eething problems) (8, 15), compatible with the dysmorphic features we observed in the proband. Vasculitis has never been described in APDS2 (although lupus-like features have) (8), but is almost certainly an autoimmune feature of APDS2 in the proband, particularly since we recently described cutaneous vasculitis in a young child with megacephaly driven by mutation in *PTEN*, another important gene in the same pathway (18).

The observation of digital vasculitis occurring in the context of absent C1q in the proband initially prompted detailed genetic scrutiny of C1q; however, routine Sanger sequencing and next generation sequencing (including WGS) excluded any mutations in *C1QA, C1QB, C1QC*, their respective promoters, or in any other complement or complement regulatory component. Moreover, mRNA expression for each of these genes was increased in the proband compatible with an attempt at increased production, as would be seen in peripheral consumption of C1q. Lastly, our *in vitro* studies indicated that monocytes from the proband could produce and release C1q, all pointing toward peripheral consumption as the cause of the absent C1q we consistently observed in serum from the proband. Hitherto, there had been no reports of C1q levels in patients with any form of APDS; we therefore contacted the authors of another report of two Spanish patients with APDS2, one of whom had the same mutation as the proband (9). These two patients were also found to have low levels of C1q, albeit to a lesser degree than our proband. Intriguingly, one of their patients (Spanish case P1, Supplemental Table 1) had modestly low levels of C1q (60 mg/L; RR 170–430), which increased transiently to be within the normal range (180 mg/L) when it was checked ~2 years after commencing sirolimus, but subsequently fell again despite adequate serum levels of sirolimus. These preliminary observations suggest that C1q consumption may be an inherent but hitherto unrecognized feature of APDS2 and is probably not permanently reversible by inhibiting mTOR (Figure 3). Nothing is yet known about C1q levels in APDS1, although, given the clinical and immunological phenotypic similarities, this is an area worthy of future study for that disease as well.

What might be the mechanism of peripheral consumption of C1q in APDS2? The answer may be suggested by considering the biology of C1q and its important regulatory role in the immune system in the context of the immunophenotype driven by constitutive PI3K activation. C1q is the first recognition subcomponent of the complement classical pathway (19). It is a 460 kDa hexameric glycoprotein, which forms a tulip-like structure, with important pattern recognition functionality, binding to various ligands including microbial surfaces, apoptotic cells, IgG and IgM, and phosphatidylserine (PS) present on apoptotic blebs and CRP, amongst other ligands (19). C1q has a major role in clearing apoptotic debris, which may otherwise drive autoimmunity (19). Indeed, genetic C1q deficiency predisposes to systemic lupus erythematosus (SLE): patients homozygous for loss of function C1q mutations have at least 90% risk of developing SLE from early in life (19). SLE is mediated by autoantibodies targeting self-antigens contained within immune complexes comprising C1q and apoptotic blebs containing nuclear antigens. These can form immune complexes that deposit in tissues, driving inflammation and the various inflammatory features of SLE (skin rash, light sensitivity, glomerulonephritis, thrombotic episodes, amongst others). We therefore speculate that the immunophenotype of APDS2 provides a “perfect storm” of immune dysregulation for C1q consumption by constitutive PI3K activation driving increased immune cellular senescence and increased apoptosis, which, in the face of dysregulated immunoglobin synthesis and elevated IgM, would result in avid C1q binding (Figure 3). Therefore, the (likely) consumptive C1q deficiency we observed in ADPS2 may represent a compensatory attempt to clear excess apoptotic debris, which, in the face of elevated IgM, results in avid C1q consumption. C1q is also highly expressed on B cells and negatively influences B cell receptor (BCR) signaling to promote tolerance (19). It may also suppress CD4^+^ T cell activation and proliferation (20), suggesting that there could be additional compensatory roles of C1q in ADPS2 designed to curtail inflammatory sequelae of constitutive PI3K activation. One limitation of our study is that we were unable to perform C1q staining from any tissue as this was not available to us.

This case illustrates that C1q consumption may represent a novel aspect of the immunophenotype associated with ADPS2 (and possibly ADPS1 as well) and again illustrates the power of next-generation sequencing to resolve complex immunophenotypes to facilitate targeted treatment and genetic counseling for families. Identification of mutated *PIK3R1* in the proband informed the therapeutic approach for this case; in addition to antibiotic prophylaxis, hydroxychloroquine, intermittent corticosteroids, and immunoglobin replacement therapy, sirolimus was added in an attempt to reduce PI3K activation via inhibition of the mammalian target of rapamycin complex (mTORC; Figure 3), although the published evidence for efficacy in ADPS2 for this approach is sparse, with an ongoing guarded prognosis for lymphoma (8). Sirolimus was not tolerated by the proband, however, with typical associated side effects manifesting (21), requiring this to be stopped. Another therapeutic alternative may be targeted PI3Kδ inhibition with leniolisib (Figure 3), and this is currently being explored in an ongoing clinical trial for ADPS (NCT02435173) (8). At the time of writing, however, the proband is currently being worked on for allogeneic hematopoietic stem cell transplantation, which is the only known curative therapeutic option (8, 22), whilst imminently receiving rituximab for chronic EBV viremia.

In conclusion, in this case report we illustrate the utility of whole genome sequencing to resolve a complex immunophenotype caused by *de novo* splicing mutation in *PIK3R1*, and we highlight consumptive C1q deficiency as a previously unrecognized component of the immunophenotype associated with APDS2. Secondly, C1q deficiency in APDS2 may further contribute to immunodeficiency and could also be associated with inflammatory immune dysregulatory phenotypes, such as the digital vasculitis observed in our case. Lastly, identification of C1q deficiency may facilitate a more rapid diagnosis if found in the context of other clinical features of APDS2 (such as hypogammaglobulinaemia or hyper IgM phenotype, short stature, facial dysmorphia and/or lymphoproliferation) by prompting targeted genetic sequencing for this specific immunodeficiency if next-generation sequencing is not readily available or is associated with long turnaround times.

## Data Availability Statement

All datasets generated for this study are included in the article/Supplementary Material.

## Ethics Statement

All experimental work was performed with ethical approval (ethics number: 08H071382) and with written informed consent from all adult participants, assent (where appropriate) for children, and parental consent for children. The proband was formally re-consented when she reached the age of 16 years. Written informed consent to participate in this study was provided by the participants' legal guardian/next of kin.

## Author Contributions

YH, SN, EO, NK, DE, and PB: design of the work, acquisition and analysis of data, drafting and revising manuscript, providing approval for publication. DE: design of the work, revising manuscript, providing approval for publication. PO, ON, KG, AW, and JL: acquisition and analysis of data, revising manuscript, providing approval for publication. CS and The Genomics England Research Consortium: oversight of methodology of whole genome sequencing, acquisition of data, revising manuscript, providing approval for publication.

### Conflict of Interest

The authors declare that the research was conducted in the absence of any commercial or financial relationships that could be construed as a potential conflict of interest.
