# Supplementary material for: Secondary C1q Deficiency in Activated PI3Kδ Syndrome Type 2
Source: Front Immunol. 2019 Nov 11;10:2589. doi: 10.3389/fimmu.2019.02589 (PMC6859795; doi:10.3389/fimmu.2019.02589)
Supplement: Supplementary file 1 [file Data_Sheet_1.pdf]

Feature (RR)	Index case (II-1)	Mother (I-1)	Father (I-2)	Asymptomatic sister (II-2)	Asymptomatic brother (II-3)	Spanish case; P1	Spanish case; P2
Age (years)	16	47	48	14	18	8	8
Gender	F	F	M	F	M	M	F
Ethnicity	Mixed	Indian	Cauc	Mixed	Mixed	Caucasian (Hispanic)	Caucasian (Hispanic)
Clinical phenotype	Many features of SHORT syndrome: IUGR, short stature, dysmorphic facial appearance with maxillary hypoplasia (Fig. 1), choanal atresia, dental abnormalities, obstructive sleep apnoea, astigmatism, gastro-oesophageal reflux, chronic diarrhoea/enteropathy	Asymptomatic; not dysmorphic	Asymptomatic; not dysmorphic	Asymptomatic; not dysmorphic	Asymptomatic; not dysmorphic	Short stature; dysmorphic facial appearance	Short stature; dysmorphic facial appearance
Clinical infections	Frequent: otitis media; sinusitis; tonsillitis; lower RTI; bronchiectasis; gingivitis; necrotising lymphadenitis (Staphylococcus aureus)	Absent*	Absent*	Absent*	Absent*	Severe bronchiolitis; recurrent lower RTI; chronic diarrhoea with recurrent Giardia lamblia and Campylobacter spp. infections. Recurrent bacterial conjunctivitis.	Recurrent suppurative cervical lymphadenitis (age 5 years), caused by Streptococcus *pneumoniae*, and S. *pyogenes*. No infections on rapamycin (target level 3-5ng/ml) and monthly immunoglobulin substitution.
Autoimmunity	Digital vasculitis; mild vitiligo; ANA^+, $DS\text{-}DNA^-$, ENA^-, RF^-, $ANCA^-$	Absent	Absent	Absent	Absent	Absent	Recurrent oral aphthous ulceration responding to short oral (<48h) corticosteroid treatment courses
Cytopenias	Absent	Absent	Absent	Absent	Absent	Absent	Absent
Lymphoproliferation	Adeno-tonsillar hypertrophy; cervical lymphadenopathy: increased number and size of lymphoid follicles, para-cortical area rich in CD3 and CD5 positive T cells, follicular hyperplasia, -ve EBV staining, no evidence of lymphoma	Absent	Absent	Absent	Absent	Generalized lymphadenopathy: reactive histological changes and predominant follicular hyperplasia; low number of plasmacytic IgG+ population; absent IgA with normal IgM. Centro-germinal B cell CD10+/bcl6+ in medullary localization. Negative EBV staining, no evidence of lymphoma	Cervical lymphadenopathy. No hepatosplenomegaly.
Pulmonary involvement	Bronchiectasis	Absent	Absent	Absent	Absent	Recurrent bronchitis	Absent
Acute phase reactants	ESR, CRP, SAA: high with infection; otherwise normal (see main text)	ESR: 2 mm/h (<10) CRP: 7 mg/L (<20)	ESR: 5 mm/h CRP: <5 mg/L	ESR: 5 mm/h CRP: <5 mg/L	ESR: 5 mm/h CRP: 11 mg/L	ESR: <10 mm/h (<10) CRP < 5mg/L (raised with infections)	ESR 5-22mm/h (<10) CRP <5mg/L (raised with infections)

**PBMCs**							
Total Lymphocyte count	2.93 X 10^9/L	N/A	N/A	N/A	N/A	3.90x10^9/L (3.00-13.5x10^9/L)	3.40x10^9/L (3.00-13.5x10^9/L)
CD3 T cell count	2.29 X 10^9/L	N/A	N/A	N/A	N/A	2.48 x10^9/L (0.85-4.3 x10^9/L)	2.67 x10^9/L (0.85-4.3 x10^9/L)
CD19 B cell count	**0.18 X10^9/L**	N/A	N/A	N/A	N/A	0.22 x10^9/L (0.39-1.4 x10^9/L)	0.35 x10^9/L (0.39-1.4 x10^9/L)
%CD4 T cell	36%	N/A	N/A	N/A	N/A	33.85%	50,0%
%CD8 T cell	41%	N/A	N/A	N/A	N/A	68.7%	45,2%
%B cell	6%	N/A	N/A	N/A	N/A	7%	10,3%
CD4:CD8	**Modestly reversed ratio**	N/A	N/A	N/A	N/A	0.49	1,1
**Immunoglobulins**							
IgG	7.41 g/L (4.9-16.1)	N/A	N/A	8.04 g/L	10.6 g/L	5.04 g/L (4.24-1.05 g/L)	7.14 g/L (4.24-1.05 g/L)
IgA	**<0.06 g/L** (0.4-2.0)	N/A	N/A	0.92 g/L	**3.36 g/L**	**0.04 g/L** (0.14-1.23 g/L)	**<0.01 g/L** (0.14-1.23 g/L)
IgM	**3.33 g/L** (0.5-2.0)	N/A	N/A	1.06 g/L	**0.38 g/L**	**6.80 g/L** (0.48-1.68 g/L)	**2.58 g/L** (0.48-1.68 g/L)
IgE	<2 kU/L (0-32)	N/A	N/A	N/A	N/A	N/A	N/A
EBV/CMV	EBV viral load: 2,180 copies/ml of whole blood (198 IU/ml); CMV not detected	N/A	N/A	N/A	N/A	EBV negative /CMV negative	EBV negative /CMV 857 (copies/ml)
Vaccine responses	Normal to tetanus and HIB	N/A	N/A	N/A	N/A	N/A	Undetectable (IgG Hib and IgG2 pneumococcus) (3yo)
**Complement**							
C1q pre sirolimus	**<13 mg/L** (50-250)*↓	140 mg/L	100 mg/L	127 mg/L	189 mg/L	**60 mg/L** (170-430)**	N/A
C1q post sirolimus	N/A	N/A	N/A	N/A		180 mg/L (170-430)	21.2 mg/L (170-430)
C2	43 mg /L (10-80)	N/A	N/A	N/A		N/A	N/A
C3c	1.43 g/L (0.75-1.65)	1.66	1.26	1.27	1.43	C3 0.97g/L (0.9-1.8)	1.73g/L (0.9—1.8)
C4	0.26 g/L (0.14-0.54)	0.55	0.26	0.18	0.42	0.38 g/L (0.1-0.4)	0.25 g/L (0.1-0.4)
Anti-C1q antibodies	<6 U/ml (0-15)	<6	<6	<6	<6	N/A	N/A
Classical complement pathway activity	**24%** (>40%)	N/A	111%	114%	N/A	N/A	N/A
Alternative complement pathway activity	99% (>10%)	N/A	77%	97%	N/A	N/A	N/A
Mannose binding lectin (RR)	3908 ng/ml (>1300)	N/A	N/A	N/A	N/A	N/A	1095 ng/ml (>1300)
HbA1c		N/A	N/A	N/A	N/A	N/A	N/A
PIK3R1 Genotype	c.1425+1G>A	WT	WT	WT	WT	c.1425+2T>A	c.1425+1G>A
Complement genotype	No mutation in any complement component,	No mutation in any complement	No mutation in any complement	N/A	N/A	N/A	N/A

	receptor or regulatory protein; no C1q promoter mutation.	component, receptor or regulatory protein no C1q promoter mutation.	component, receptor or regulatory protein no C1q promoter mutation.				
Treatment (past and **present**)	Prednisolone; intravenous methylprednisolone; **hydroxychloroquine**; azathioprine; amlodipine; antibiotic (**prophylaxis; and full therapeutic courses for acute infections**); **IG replacement therapy**; sirolimus; adenotonsillectomy; multiple grommet insertions for otitis; CPAP for obstructive sleep apnoea; Nissen's fundoplication; planned future facial reconstruction for orthodontic issues	N/A	N/A	N/A	N/A	Initial prophylaxis with TMP-SFX and IVIG. Intermittent metronidazole for GI infections, growth hormone (stopped after genetic diagnosis). **Sirolimus 1mg/m^2/day, IG 0.5g s.c. monthly, antibiotic prophylaxis with Amoxicillin 30mg/kg/72h, topical tobramycin eye drops**	Initial prophylaxis with TMP-SFX and IVIG, intermittent metronidazole for GI infections, **Sirolimus 0.8mg/m^2, IG 0.5g s.c. monthly, off antibiotic prophylaxis**<br><br>**NOTE: if not stated otherwise laboratory results were taken at genetic diagnosis (5 years of age)**

Supplemental Table 1: Clinical and laboratory features of pedigree, and other previously published case.

Numeric results in bold indicate abnormality. SHORT syndrome: Short stature, hyperextensibility, hernia, ocular depression, and teeth abnormalities; HbA1c, glycosylated haemoglobin; ↓Low C1q confirmed on 5 separate occasions, over a 14-year period; N/A, not assessed, or not applicable; PBMCs, peripheral blood mononuclear cells; ANA, antinuclear antibody 1:640; DS-DNA, autoantibodies to double-stranded deoxyribonucleic acid; ENA, autoantibodies to extractable nuclear antigens; RF, rheumatoid factor; ANCA, anti-neutrophil cytoplasmic antibodies; Cauc, Caucasian; F, female; M, male; RR, reference range; EBV, Epstein-Barr virus; IU, international units; WT, wild type; HIB, Haemophilus Influenza B; ESR, erythrocyte sedimentation rate; CRP, C-reactive protein; SAA, serum amyloid A; RTI, respiratory tract infection; TMP-SFX, trimethoprim- sulphamethoxazole; IVIG, intravenous immunoglobin; GI, gastrointestinal; IG, immunoglobin; s.c., subcutaneously. * C1q assay using ELISA, ** C1q assay using nephelometry

Supplemental Table 2: sequences and position on chromosome 1 of the forward and reverse primers used for Sanger sequencing of all the exons and untranslated regions of *C1qA, C1qB* and *C1qC.*

Gene	Forward sequence	Reverse sequence	Start position*	End position*
C1qA	GTTCACCATGTGCCAAGTGT	GTGGCCAAGTTTCACATCCC	22,636,075	22,636,597
	AATGTCCCTGGTGAGCTTCTG	ACAGGAATTCACAAACGCCC	22,636,528	22,637,118
	CGCTTTGGGCGTTTGTGAAT	CCTGATTCTCAGTGCCACCC	22,637,112	22,637,849
	GGGAAGAGGTCCTGACCAAAG	GTAGTAGCCGGGTACAGTGC	22,638,368	22,639,127
	TCCCGGGAATTAAAGGCACC	AAGGACGGGCCTGACTCTTA	22,638,956	22,639,674
	CCCCGTGACACATGCTCTAA	TGAGGCTGCTAGGACATGCT	22,639,532	22,640,033
	TCTTCCCTTCTGTTCAACCAC	TGTTAAGAGGCCACTGTACCTG	22,654,488	22,654,934
Exon 1			22,636,506	22,636,702
Exon 2			22,637,610	22,637,779
Exon 3			22,638,833	22,639,608
C1qB	ATGCTCACTGAGGAACACCC	CCCAGCTACCTGCACTGTTA	22,652,909	22,653,416
	GGCAGAAAGTGGTGATTCCG	AACTCTACCTGCACATCCCC	22,653,350	22,653,860
	TATAAGCCAGTCGCTCCTGC	GGAGTGGGAAAGTGCTGGTT	22,653,759	22,654,297
	CCACGGTGGTAACCTCTCAC	TTGAACCCCATGACAACCCC	22,659,404	22,660,054
	CTGAAGAAGGACCCCCACAC	TGGTGATCACGTGGTCGAAG	22,660,523	22,661,066
	ATCGGGAGACTACAAGGCCA	AGGTGTAGGCTACCACTGGG	22,660,960	22,661,724
	AAGGTGGCCCGTTCATAAGC	CCTCTATTCCTCCATCTGTCCC	22,658,092	22,658,705
Exon 1			22,653,189	22,653,303
Exon 2			22,659,440	22,659,643
Exon 3			22,660,812	22,661,536
C1qC	GCAGGGGACAGTTCCTCATT	CAACCCTCTCATTCCCCAGG	22,643,431	22,643,962
	CTCTGACCACTCAGACACCG	ACCGTGAGAGTCCACCTCTG	22,643,621	22,644,349
	AAAGCCTAACTCCCTGCACC	CGACGCGTGGTAGACAAAGT	22,647,065	22,647,564
	CCAACCCGCAGGGAGATTAT	AGCACCAACCAGATGCCTTG	22,647,479	22,648,211
Exon 1			22,643,633	22,643,737
Exon 2			22,644,011	22,644,204
Exon 3			22,647,227	22,648,110

Positions are based on Genome reference consortium human build 38 patch 7 (GRCh38.p7)

Supplemental Table 3: Sequences of forward and reverse primers used for Sanger sequencing of all cDNA exons and untranslated regions of *C1qA, C1qB* and *C1qC*

	Forward sequence	Reverse sequence
C1QA	TGGAGTTGACAACAGGAGGC	CGGGTACAGTGCAGACGAAT
	GCAACGTGGTCATCTTCGAC	CCCCTTGAGGAGGAGACGAT
	CTGGGCTTCTGTGACACCAC	ATTTTACAGGCGGAGCATGGA
C1QB	CTTCCCAGGAGGCGTCTGA	GTGTTGGGGGTAGAGTGAGC

C1QC	TGTGCCAGGCCAGAAACC	TAGTCGGGGAAGAGCAGGAA

Supplemental Table 4: Sequences of forward and reverse primers used for qPCR of C1qA, C1qB, C1qC and β-actin

	Forward	Reverse
B-actin	GCAATGAGCGGTTCCGCTGC	CGATCCACACGGAGTACTTG
C1QA	TGGAGTTGACAACAGGAGGC	CGATATGGCCAGCACACAGA
C1QB	GACCGAGGGCAGTAGGCTC	TCATCATACTGTGTCAGACGCC
C1QC	AAGGATGGGTACGACGGACT	GTAAGCCGGGTTCTCCCTTC

Supplemental table 5: Vasculitis and inflammation panel gene list

**Gene ID**	**Gene name**	**Transcript**
ACP5	Acid phosphatase-5/tartrate-resistant phosphatase	NM_001111034
ACTA2	Actin alpha 2	NM_001613
ADA2	Cat eye syndrome chromosome region 1/Adenosine deaminase 2	NM_001282225
ADAM17	ADAM Metallopeptidase Domain 17	NM_003183
ADAR	Adenosine deaminase acting on RNA	NM_001111
AICDA	Activation-induced cytidine deaminase	NM_020661
AIRE	Autoimmune Regulator	NM_000383
AP1S3	Adaptor Related Protein Complex 1 Sigma 3 Subunit	NM_001039569
AP3B1	Adaptor Related Protein Complex 3 Beta 1 Subunit	NM_003664
APOA1	Apolipoprotein A1	NM_000039
APOA2	Apolipoprotein A2	NM_001643
APOA4	Apolipoprotein A4	NM_000482
APOC2	Apolipoprotein C2	NM_000483
APOC3	Apolipoprotein C3	NM_000040
APOE	Apolipoprotein E	NM_000041
APP	Amyloid Beta Precursor Protein	NM_000484
ARPC1B	Actin Related Protein 2/3 Complex Subunit 1B	NM_005720
B2M	Beta-2-Microglobulin	NM_004048
BLOC1S6	Biogenesis Of Lysosomal Organelles Complex 1 Subunit 6 (BLOC1S6)	NM_012388
BMPR2	Bone morphogenetic protein type II receptor	NM_001204
BTK	Bruton's tyrosine kinase	NM_000061
C1QA	Complement C1q A Chain	NM_015991
C1QB	Complement C1q B Chain	NM_000491
C1QC	Complement C1q C Chain	NM_172369
C1R	Complement C1r	NM_001733
C2	Complement C2	NM_000063

C3	Complement C3	NM_000064
C5	Complement C5	NM_001735
C6	Complement C6	NM_000065
C7	Complement C7	NM_000587
C8A	Complement C8 Alpha Chain	NM_000562
C8B	Complement C8 Beta Chain	NM_000066
C9	Complement C9	NM_001737
CARD14	Caspase Recruitment Domain Family Member 14	NM_024110
CASP10	Caspase 10	NM_032977
CASP8	Caspase 8	NM_033355
CBL	Cbl Proto-Oncogene, E3 Ubiquitin Protein Ligase	NM_005188
CBS	Cystathionine beta synthase	NM_000071
CD40LG	CD40 antigen ligand	NM_000074
CD70	Tumor Necrosis Factor Ligand Superfamily Member 7	NM_001252
CFB	Complement Factor B	NM_001710
CFD	Complement Factor D	NM_001928
CFH	Complement factor H	NM_000186
CFHR5	Complement factor H-related protein 5	NM_030787
CFI	Complement factor 1	NM_000204
CFP	Complement Factor Properdin	NM_002621
COL3A1	Collagen Type III Alpha 1 Chain	NM_000090
COL4A1	Collagen Type IV Alpha 1 Chain	NM_001845
COL5A1	Collagen Type V Alpha 1 Chain	NM_000093
COL5A2	Collagen Type V Alpha 2 Chain	NM_000393
COL7A1	Collagen Type VII Alpha 1 Chain	NM_000094
COPA	Coatomer subunit alpha	NM_001098398 NM_004371
CORO1A	Coronin, actin binding protein, 1A	NM_007074
CPT2	Carnitine palmitoyltransferase 2	NM_000098
CST3	Cystatin C3	NM_000099
CTC1	CTS telomere maintenance complex component 1	NM_025099
CTLA4	Cytotoxic T-Lymphocyte Associated Protein 4	NM_005214
CTPS1	Cytidine 5’ triphosphate synthase 1	NM_001905
CYBA	Cytochrome b alpha chain	NM_000101
CYBB	Cytochrome b beta chainp91-phox	NM_000397
DCLRE1C	DNA cross-link repair 1c	NM_001033855
DNASE1	Deoxyribonuclease 1	NM_005223
DNASE1L3	Deoxyribonuclease I-like 3	NM_004944
DNASE2	deoxyribonuclease II, lysosomal	NM_001375
DOCK8	Dedicator of cytokinesis 8	NM_203447
DYSF	Dysferlin	NM_001130987

EFEMP2	EGF-containing fibulin-like extracellular matrix protein 2; also referred to as Fibulin 4; FBLN4	NM_016938
ELANE	Elastase, neutrophil-expressed	NM_001972
ELN	Elastin	NM_001278939
FAS	Tumour necrosis factor receptor superfamily member 6	NM_000043
FASLG	Tumor necrosis factor ligand superfamily member 6 (FAS ligand)	NM_000639
FBLIM1	Filamin binding lim protein 1	NM_017556
FBN1	Fibrillin 1	NM_000138
FBN2	Fibrillin 2	NM_001999
FERMT1	Ferritin family member 1	NM_017671
FGA	Fibrinogen Alpha Chain	NM_000508
FLNA	Filamin A	NM_001456
FOXE3	Forkhead Box E3	NM_012186
FOXP3	Forkhead box P3	NM_014009
G6PC3	Glucose-6-phosphatase 3	NM_138387
GATA2	GATA-binding protein 2	NM_032638 NM_001145661
GLA	Alpha-galactosidase A	NM_000169
GSN	Gelsolin	NM_001127662
GUCY1A1	Guanylate Cyclase 1 Soluble Subunit Alpha 1	NM_000856
GUCY2C	Guanylate cyclase 2C	NM_004963
HFE	hemochromatosis	NM_000410
HPS1	Hermansky-Pudlak syndrome type 1	NM_000195
HPS4	Hermansky-Pudlak syndrome type 4	NM_022081
HPS6	Hermansky-Pudlak syndrome type 6	NM_024747
HTR1A	5-Hydroxytryptamine Receptor 1A	NM_000524
HTRA1	HtrA serine peptidase-1 gene	NM_002775
ICOS	Inducible T-cell co-stimulator	NM_012092
IFIH1	Interferon-induced helicase C domain-containing protein 1	NM_022168
IFNGR1	Interferon gamma receptor 1	NM_000416
IFNGR2	Interferon gamma receptor 2 (interferon gamma transducer 1)	NM_005534
IKBKG	IKK-gamma	NM_003639
IL10	Interleukin 10	NM_000572
IL10RA	Interleukin 10 receptor, alpha	NM_001558
IL10RB	Interleukin 10 receptor, beta	NM_000628
IL1RN	Interleukin 1 receptor antagonist	NM_173842
IL2RA	Interleukin 2 receptor, alpha chain	NM_000417
IL31RA	Interleukin 31 Receptor A	NM_139017
IL36RN	Interleukin 36 receptor antagonist	NM_173170
IRF8	Interferon Regulatory Factor 8	NM_002163
ISG15	ISG15 Ubiquitin-Like Modifier	NM_005101

ITGB2	Beta-2 integrin chain	NM_000211
LACC1	Laccase domain containing 1	NM_001128303
LMNA	Laminin A	NM_170707
LOX	Lysyl Oxidase	NM_001178102<br>NM_001317073<br>NM_002317
LPIN2	Lipin 2	NM_014646
LRBA	Lipopolysaccharide-responsive and beige-like anchor brotein	NM_001199282
LYN	Tyrosine-Protein Kinase	NM_002350
LYST	Lysosomal trafficking regulator	NM_000081
LYZ	Lysozyme	NM_000239
MAGT1	Magnesium Transporter 1	NM_032121
MASP2	Mannose-binding lectin serine protease 2	NM_006610
MAT2A	Methionine Adenosyltransferase 2A	NM_005911
MBL2	Mannose-binding lectin	NM_000242
MEFV	MEditerranean FeVer	NM_000243
MFAP5	Microfibrillar Associated Protein 5	NM_003480
MVK	Mevalonate Kinase	NM_000431
MYD88	Myeloid Differentiation Primary Response 88	NM_001172569<br>NM_002468
MYH11	Myosin, Heavy Chain 11, Smooth Muscle	NM_001040113
MYLK	Myosin Light Chain Kinase	NM_053025
NCF2	Neutrophil cytosol factor 2	NM_000433
NCF4	Neutrophil cytosol factor 4	NM_000631
NF1	Neurofibromin 1	NM_000267
NFKB1	Nuclear Factor Kappa B Subunit 1	NM_003998
NLRC4	NLR Family CARD Domain Containing 4	NM_021209
NLRP1	NLR Family Pyrin Domain Containing 1	NM_033004
NLRP12	NLR Family Pyrin Domain Containing 12	NM_144687
NLRP3	NLR Family Pyrin Domain Containing 3	NM_001243133
NLRP6	NLR Family Pyrin Domain Containing 6	NM_138329
NLRP7	NLR Family Pyrin Domain Containing 7	NM_001127255
NOD2	Nucleotide-binding oligomerization domain 2	NM_022162
NOTCH1	Notch 1	NM_017617
NOTCH3	Notch 3	NM_000435
NRAS	Neuroblastoma ras	NM_002524
OSMR	Oncostatin M Receptor	NM_001323505<br>NM_001323506<br>NM_003999
OTULIN	OUT deubiquitinase with linear linkage specificity	NM_138348
PIK3CD	Phosphatidylinositol-4,5-bisphosphate 3-kinase, catalytic subunit delta	NM_005026
PIK3R1	Phosphatidylinositol 3-kinase regulatory subunit alpha	NM_181504

PLCG2	Phospholipase C, Gamma-2	NM_002661
PLOD1	Procollagen-lysine, 2-oxoglutarate 5-dioxygenase 1	NM_001316320
POMP	Proteasome maturation protein	NM_015932
PRF1	Perforin	NM_005041
PRG4	Proteoglycan 4	NM_005807
PRKCD	Protein Kinase C, Delta	NM_006254
PRKG1	Protein kinase, cGMP-dependent, type I	NM_001098512
PSMA3	Proteasome Subunit Alpha 3	NM_002788
PSMB4	Proteasome Subunit Beta 4	NM_002796
PSMB8	Proteasome Subunit Beta 8	NM_148919
PSMB9	Proteasome Subunit Beta 9	NM_002800
PSTPIP1	Proline-Serine-Threonine Phosphatase Interacting Protein 1	NM_003978
PTEN	Phosphatase and tensin homolog	NM_000314<br>NM_001304717
PYCARD	PYD and CARD domain containing	NM_013258
RAB27A	RAB27A, Member RAS Oncogene Family	NM_004580
RAG1	Recombinant activating gene 1	NM_000448
RAG2	Recombination Activating 2	NM_000536
RANBP2	RAN Binding Protein 2	NM_006267
RASGRP1	RAS Guanyl Releasing Protein 1	NM_001128602<br>NM_005739
RBCK1	RANBP2-Type And C3HC4-Type Zinc Finger Containing 1	NM_031229
RET	Ret Proto-Oncogene	NM_020975
RHOD	Ras Homolog Family Member D	NM_014578
RNASEH2A	Ribonuclease H2 subunit A	NM_006397
RNASEH2B	Ribonuclease H2 subunit B	NM_024570
RNASEH2C	Ribonuclease H2 subunit C	NM_032193
RNF213	Ring Finger Protein 213	NM_001256071
RNF31	Ring Finger Protein 31	NM_017999
SAMD9L	Sterile alpha motif domain containing 9 like	NM_001303500
SAMHD1	SAM-domain and HD-containing protein 1	NM_015474
SCN9A	Sodium channel, voltage-gated, type IX, alpha subunit	NM_002977
SERPING1	Serpin Peptidase Inhibitor, Clade G (C1 Inhibitor), Member 1	NM_000062
SH2D1A	SH2-domain protein 1a (Slam-associated protein)	NM_002351
SH3BP2	SH3-domain binding protein 2	NM_003023
SKI	v-ski avian sarcoma viral oncogene homolog	NM_003036
SKIV2L	Superkiller viralicidic activity 2-like	NM_006929
SLC29A3	Solute carrier family 29 (nucleoside transporter), member 3	NM_018344

SLC2A10	Solute carrier family 2 (facilitated glucose transporter), member 10	NM_030777
SLC37A4	Solute carrier family 37 (glucose-6-phosphate transporter), member 4	NM_001467
SLC7A7	Solute Carrier Family 7 Member 7	NM_001126106
SMAD2	SMAD Family Member 2	NM_005901
SMAD3	SMAD family member 3	NM_005902
SMAD4	SMAD family member 4	NM_005359
SNORD118	Small Nucleolar RNA, C/D Box 118	NR_033294
STAT3	Signal Transducer And Activator Of Transcription 3	NM_139276
STK4	Serine/Threonine Kinase 4	NM_006282
STX11	Syntaxin 11	NM_003764
STXBP2	Syntaxin binding protein 2	NM_006949
TGFB2	transforming growth factor, beta 2	NM_001135599
TGFB3	Transforming growth factor, beta-3	NM_003239
TGFBI	Transforming Growth Factor Beta Induced	NM_000358
TGFBR1	Transforming growth factor-beta receptor, type 1	NM_004612
TGFBR2	Transforming growth factor-beta receptor, type 2	NM_001024847
TMEM173	Transmembrane protein 173	NM_198282
TNFAIP3	TNF Alpha Induced Protein 3	NM_001270507
TNFRSF11A	TNF Receptor Superfamily Member 11a	NM_003839
TNFRSF1A	TNF Receptor Superfamily Member 1A	NM_001065
TRAP1	TNF Receptor Associated Protein 1	NM_016292
TREX1	Three prime repair exonuclease 1	NM_016381
TRIM28	Tripartite Motif Containing 28	NM_005762
TRNT1	tRNA nucleotidyl transferase, CCA-adding, 1	NM_182916
TTC37	Tetratricopeptide repeat domain 37	NM_014639
TTR	Transthyretin	NM_000371
TYK2	Tyrosine Kinase 2	NM_003331
UNC13D	Unc-13 Homolog D	NM_199242
USB1	U6 SnRNA Biogenesis Phosphodiesterase 1	NM_024598
USP18	Ubiquitin-specific protease 18	NM_017414
VPS13B	Vacuolar protein sorting 13 homolog B (yeast)	NM_017890
WAS	Wiskott-Aldrich syndrome	NM_000377
WDR1	WD Repeat containing 1/Actin-interacting protein 1	NM_005112
XIAP	X-linked inhibitor of apoptosis	NM_001167
YY1AP1	YY1 Associated Protein 1	NM_001198906 NM_139118
ZAP70	Zeta Chain Of T Cell Receptor Associated Protein Kinase 70	NM_001079

Supplemental Table 6: T cell immunophenotype of proband

Naive:CD4+CD45RA+CD27+	11.8 %
Memory:CD4+CD45RA-CD27+	69.2 %
Effector:CD4+CD45RA+CD27-	0.0 %
Naive:CD8+CD45RA+CD27+	18.4 %
Memory:CD8+CD45RA-CD27+	58.5 %
Effector:CD8+CD45RA+CD27-	6.2 %

Naive T cells present but in a low proportion for age (see main text).

Supplemental methods

PBMC isolation and monocyte-derived macrophage cell culture

Peripheral blood mononuclear cells (PBMCs) were isolated from 5ml of blood by density gradient centrifugation (Lymphoprep; StemCell Technologies, Inc.) and cultured in RPMI1640 medium with glutamine (Sigma-Aldrich) supplemented with 10% FCS. 2 x 10^6 PBMC were plated in a 24 well plate and after 2 hours, adherent monocytes were washed and cultured in presence of 100 ng/ml of M-CSF (Peprotech) for 5 days in order to differentiate them to macrophages (MDM).

Cell stimulation

Adherent monocytes or monocyte-derived macrophages were stimulated with 10 µM dexamethasone and 200 ng/ml IFN-γ as described before to increase the C1q production (1). After 72 hours, the supernatant and the cells were collected. The supernatants were used for ELISA and the cells for immunostaining and flow cytometry analysis of C1q expression.

Detection of C1q production in macrophages by immunofluorescence microscopy

2 x 10^5 macrophages from controls and patient were seeded on polylysine-coated coverslip (ThermoFisher Scientific) in a 24 well plate and left to adhere for 2 hours. To simulate MDM to produce C1q, cells were stimulated with 10µM dexamethasone and 100 ng/ml IFN-γ for 72 hours. Cells were fixed with 4% paraformaldehyde and permeabilised with 1% Triton. Cells were incubated with sheep polyclonal anti-human C1q (1:100, IgG fraction; Binding Site) for 2 hours, following with Alexa Fluor 488 donkey anti-sheep IgG at RT for 1 hour. Lastly, the coverslips were put on slides with Vectashield antifade mounting medium with DAPI (Vector Laboratories) and sealed with nail-varnish. Images were captured using a Zeiss LSM 710 inverted confocal microscope (Zeiss) and analyzed with ImageJ (National Institute of Health, USA).

Detection of C1q in peripheral blood mononuclear cells by flow cytometry

Cells were stained with FITC-conjugated goat anti-human C1q (DAKO) and the expression of C1q on monocyte cell surface was analysed using flow cytometry (FACS Calibre); to measure the intracellular expression of C1q, cells were first permeabilised using Fixation/Permeabilisation solution (Fix/Perm kit, BD), then stained with C1q for flow cytometry analysis.

Detection of C1q by ELISA and nephelometry

C1q levels in culture supernatants were analysed by ELISA (ThermoFisher Scientific), following manufacturer instructions. The serum level of C1q was measured by ELISA (GOSH) or nephelometry (Spain). The normal range for C1q is 50-250 mg/L by ELISA or 170- 430 mg/L by nephelometry.

Whole Genome sequencing

Targeted panel next generation sequencing and Sanger sequencing

Targeted panel next generation sequencing of the index case (II-1) was performed using the Vasculitis and AutoInflammation Panel (VIP) (2). Briefly, the Agilent eArray online tool (https://earray.chem.agilent.com/suredesign/) was used to design the VIP NGS gene panel that targeted 214 genes associated with monogenic autoinflammatory diseases, including complement genes (supplemental Table 5). Captured and indexed libraries (QXT Target Enrichment System) were sequenced as a multiplex of 16 samples on an Illumina MiSeq sequencer in paired-end mode. Read alignment, variant calling, and annotation were performed using Agilent Sure Call v 4.0.1.46 software.

Sanger sequencing was performed on genomic DNA obtained from all 5 family members, using the primers 5' – GATGTTTCCATGTCAGCTATTTTG –3' and 5'– ACAAATAAATGCTCTCACCCC– 3' to amplify exon 11 of *PI3KR1* including the adjacent splice sites. The amplified product was sequenced with the Big Dye Terminator v3.1 Cycle sequencing kit (Applied Biosystems, Foster City, CA, USA) and subsequently analyzed on the ABI 3730 DNA analyzer system.

Supplementary references

1. Walker DG. Expression and regulation of complement C1q by human THP-1-derived macrophages. Molecular and chemical neuropathology. 1998;34(2-3):197-218.

2. Omoyinmi E, Standing A, Keylock A, Price-Kuehne F, Melo Gomes S, Rowczenio D, et al. Clinical impact of a targeted next-generation sequencing gene panel for autoinflammation and vasculitis. PLoS ONE [Internet]. 2017 Jul 27 [cited 2018 Apr 17];12(7). Available from: https://www.ncbi.nlm.nih.gov/pmc/articles/PMC5531484/
